# Investigation and analysis of standardized training for residents of general practitioners of Gansu Province in China

**DOI:** 10.1186/s12875-020-01185-y

**Published:** 2020-06-19

**Authors:** Hongjing Wang, Jin He, Dongzhi Zhang, Yue Wu, Peidong Wang, Hui Cai

**Affiliations:** 1 Department of Medical Affairs, Gansu Provincial Hospital, Lanzhou, Gansu China; 2 Department of Discipline Construction Management, Gansu Provincial Hospital, Lanzhou, Gansu China; 3 Department of Burn, Gansu Provincial Hospital, Lanzhou, Gansu China; 4 Department of Residents Training Management, Gansu Provincial Hospital, Lanzhou, Gansu China; 5 Gansu Provincial Hospital, Lanzhou, Gansu China; 6 Hospital Management Research Center, Lanzhou University, Lanzhou, Gansu China

**Keywords:** Standardized training, General practitioners, China

## Abstract

**Background:**

China’s standardized training for residents of General Practitioners (GPs) is aimed at providing the postgraduate training for family doctors who will serve the primary health care institutions. The aim of this paper is to investigate the standardized training situation, satisfaction with standardized training, work situation, intention, satisfaction and attitude of GPs who have finished standardized training.

**Methods:**

This study was undertaken in 6 training hospitals in Gansu province using a questionnaire with 45 questions.

**Results:**

Approximately 275 residents of GPs were enrolled. Finally, 263 residents completed the questionnaire (95.64% response rate), including 133 females (50.57%) and 130 males (49.43%) with an average age of 28 years (standard deviation, 1.93 years; range, 25–36 years). Additionally, 56.65% were single and 43.35% were married. Of all subjects, 92.40% residents had obtained certification of standardized training for residents of GPs and only 39.54% residents were satisfied with monthly income during training. There were 171 oriented rural medical graduates, of whom, only 42.69% expressed the willing to continue working in the primary health care institutions after the serve time (6 years) expired. Around 86.31% of residents of GPs who had finished standardized training got jobs with more than half serving in the primary health care institutions. For medical institutions and sanitary bureau were clear about general medicine policies, only 29.96% subjects registered as GPs. Among the residents in general practice department, 68.42% were engaged in the diagnosis and treatment of common disease and frequently-occurring diseases as well as referral of patients. The percentage of residents who were satisfied with the job and income were 30.40 and 14.98%, respectively.

**Conclusion:**

Standardized training for residents of GPs in China is gradually improving. In order to cultivate more GPs and increase their willing to serve in the primary health care institutions, it is necessary to formulate and execute better policy of GPs, publize general medicine and improve the training quality.

## Background

China is a developing country with a population of more than 1.3 billion. With the development of society’s economy and the improvement of the living level, people are demanding higher health care. At the same time, industrialization, urbanization and ecological environment changes more and more factors affecting health, population aging and changes in disease spectrum also put new demands on medical and health services. In the metropolis, difficulties in seeing a doctor and higher cost of seeing a doctor come from skip-level diagnosis and treatment. And this phenomenon stems from the weakness of the ability of primary medical institutions which leads to people’s distrust of primary medical institutions. The construction of basic medical and health personnel team in China lags behind, and the number of qualified general practitioners (GPs) is insufficient, which restricts the improvement of basic medical and health services [1].

According to the 2018 China Statistical Yearbook, Gansu Province has 26,579 primary health care institutions, 199,155 health workers, and 21,358 rural health workers, lower than the average level in China (30,097, 378,673 and 31,331) [2]. The differential analysis on primary health care capacity showed regional differences among 31 regions in China with medical human resources of primary health care institutions in Gansu Province ranking at 16 [3]. In Gansu, the number of doctors per 1000 people is 2.26, below the average level in China (2.59). And the number of GPs per 10,000 people is only 2.58, far from targeted number. So, qualified GPs of Gansu is inadequate.

A recent study from Stanford medical school and Harvard medical school proved that increasing primary care physicians resulted in decreased mortality. GPs are the main force of the medical team, and also the first doctor to visit when patients seek help for common diseases in American [4]. Barbara Starfield indicated that primary care contributed to prevent illness and death and primary care was associated with a more equitable health distribution among populations, and, therefore, health promoting value of primary care contributed a lot in health care system [5]. China has paid attention to primary care and the training and development of GPs. The standardized training system for residents has been tested in some provinces and cities, such as Sichuan, Shanhai and Beijing [6–8]. So far, this system is well implemented and resident physicians enjoy an improvement in clinical ability [9, 10]. In Dec 2013, guidance on the establishment of standardized training system for residents was published by the National Health Commission of the People’s Republic of China and other 7 departments. The standardized training system included 34 majors, including general medicine. According to the guidance, all provinces will comprehensively launch standardized training for residents by 2015, and a standardized training system for residents will be established by 2020. All medical clinicians with bachelor degree and above will receive standardized training for residents [11].

In China, GPs are called gatekeepers to the health of the inhabitants, who are comprehensive medical talents and undertake prevention, diagnosis and treatment of common and frequently-occurring diseases, referral, rehabilitation, chronic disease management and health management in the primary health care institutions. Therefore, the State Council issued opinions on the reform and improvement of cultivation and incentive mechanism for GPs [12]. Establishing a hierachical medical system and implementing a GP contract service, and the responsibility system for medical and health services to individual doctors are the development direction of China’s medical and health services. By 2020, an energetic GP system will be established in China, and a unified and standardized GP training model, also the “first-time in primary health care institutions” service model will be formed. The goal of two to three qualified GPs per 10,000 inhabitants in urban and rural areas will be achieved. The service level of GPs will be improved to requirements of the basic medical and health service needs of the people. However, there were only 253,000 qualified GPs in China and only 1.81 GPs per 10,000 populations by the end of 2017, far below the health needs of the people [13].

In 2010, the National Development and Reform Commission and other departments initiated the policy of free training for rural order-oriented medical students, in which, students could enjoy tuition-free education and subsistence allowance in the medical university. They should sign targeted employment agreements with the school, health commission and human resources society before getting admission notices and promise to serve in the rural primary health care institutions for 6 years after graduation. And they will be cultivated according to the GP requirements [14]. After reporting for duty, they participate in the three-year standardized training for residents of GPs, which is included in the six-year service period. During this period, they will be educated for general medicine primary knowledge, primary operational skills and especially dialectic thinking of clinical diagnosis and treatment. It includes 2 years and a half in hospital and 6 months in primary health care institutions with GP clinics. At present, the standardized training for rural order-oriented medical graduates is the main training method of GPs [15].

Since the implementation of the standardization training for GPs in Gansu Province in 2014, there have been two cohorts GP residents graduated. Therefore, it is necessary to investigate the training situation, work status and satisfaction to improve the training of GPs.

## Methods

### Questionnaire formulation

The questionnaire was discussed and formulated by experts including the administrative managers, clinical directors and clinical teachers in Gansu Provincial Hospital. A pilot-test was conducted in Gansu Provincial Hospital, and the questionnaire was evaluated for the reliability (Cronbach’s alpha coefficient was 0.91) and validity (Goodness of Fit Index was 0.87). After the further modifications and revisions, the final questionnaire included 45 questions, concerning basic personal information (8 items), standardized training situation (9 items), satisfaction of standardized training (6 items), work situation and intention (13 items) and satisfaction of work and attitude for GPs (9 items).

### Sample size and method

The research team reviewed all GPs who had finished standardized training in 6 training hospitals in 2017 and 2018, for the three-year standardized training for residents was fully initiated in 2014 in Gansu. Finally, 275 respondents met the requirements of the survey, and 273 completed questionnaires. Gansu Provincial Hospital was the quality control center for general medicine of Gansu, facilitating the investigation and research. A researcher was responsible for distributing the questionnaire and training hospitals directors and then collecting them. The study used an anonymous self-reporting method. All subjects provided written informed consents.

### Statistical analysis

Invalid questionnaires were excluded and the data were analyzed in Excel with descriptive statistics (number and percentage).

## Results

### Basic characteristics

Approximately 275 residents of GPs were investigated. Finally, 263 residents were available (95.64%) including 133 (50.57%) women and 130 (49.43%) men with an average age of 28 years with a *SD* of 1.93 (Table 1). Of these subjects, 114 (43.35%) were married and 149 (56.65%) were single. There were 2 (0.76%) with junior college degree, 240 (91.25%) with bachelor degree and 21 (7.98%) with master degree. The proportion of residents completing the standard training were 17.87% (*n* = 47) in 2017 and 82.13% (*n* = 216) in 2018, respectively. Among all the 6 training hospitals, 59 (22.43%) were trained in Gansu Provincial Hospital, 96 (36.50%) in The First Hospital of Lanzhou University, 57 (21.67%) in The Second Hospital of Lanzhou University, 41 (15.59%) in Chinese People’s Liberation Army 940 Hospital, 6 (2.28%) in The First Hospital of Tianshui and 4 (1.52%) in The People’s Hospital of Pingliang. The study included 171 (65.02%) rural oriented medical graduates. There were 224 (85.17%) fresh graduates and 39 (14.83%) former graduates.

**Table 1:** Demographic characteristic of the respondents

Demographic characteristic		Frequency	Percent (%)
Gender	Male	130	49.43
	Female	133	50.57
Age	<=25	7	2.67
	25–30	223	84.79
	> 30	33	12.54
Marital status	Married	114	43.35
	Unmarried	149	56.65
Education	Junior college degree	2	0.76
	Bachelor degree	240	91.25
	Master degree	21	7.98
Years of accepting training	2014–2017	47	17.87
	2015–2018	216	82.13
Residents of training hospital	Gansu Provincial Hospital	59	22.43
	The First Hospital of Lanzhou University	96	36.50
	The Second Hospital of Lanzhou University	57	21.67
	Chinese People’s Liberation Army 940 Hospital	41	15.59
	The First Hospital of Tianshui	6	2.28
	The People’s Hospital of Pingliang	4	1.52
Residents type	Rural oriented medical graduates	171	65.02
	Other graduates	92	34.98
Graduate type	Fresh graduates	224	85.17
	Former graduates	39	14.83

### General situation of standardized training for residents of GPs

A total of 243 (92.40%) residents passed certification exam (Table 2), of whom, 180 (68.44%) considered it necessary for the standardized training whereas 20 (7.60%) thought it unnecessary. More than half of residents’ monthly income was among 2000 and 3000RMB. Only 101(38.40%) residents expressed improvement in the comprehensive ability by standardized training, while 77 (29.80%) thought the training useless. The residents of GPs were improved mainly in clinical operational skills (91.63%) and medical theoretical knowledge (71.48%). Only 84 (31.94%) residents of GPs deemed that their income were higher than other specialties. Of the rural oriented medical graduates, 73 (42.69%) expressed the willing to continue to work in the primary health care institutions after the rural serve time was finished and more graduates (57.31%) had no willing. Those who didn’t want to work in the primary health care institutions planned to take part in the graduate candidate test (69.39%) or find a better position (74.49%).

**Table 2:** The situation of standardized training for residents of GPs

Statements	Frequency	Percent (%)
Whether you obtain certification of the standardized training for residents of GPs. (*n* = 263)		
Yes	243	92.40
No	20	7.60
Is it necessary to launch the standardized training for residents of GPs. (*n* = 263)		
Necessary	180	68.44
Neutral	63	23.95
Non- necessary	20	7.60
Monthly income during the standardized training for residents of GPs (RMB). (*n* = 263)		
< =2000	76	28.90
2000–3000	167	63.50
3000–4000	17	6.46
> 4000	3	1.14
Is it useful to improve comprehensive ability of residents of GPs by standardized training. (*n* = 263)		
Yes	101	38.40
A little	85	32.32
No	77	29.28
The abilities of residents of GPs are improved by standardized training. (*n* = 263)		
Medical ethics	81	30.80
Medical theoretical knowledge	188	71.48
Clinical operational skills	241	91.63
Sociability	129	49.05
Scientific research ability	60	22.81
The income of GPs is higher than other specialties. (*n* = 263)		
Yes	84	31.94
No	179	68.06
As a rural oriented medical graduate, whether you have the willing to continue to work in the primary health care institutions after the rural serve is finished. (*n* = 171)		
Yes	73	42.69
No	98	57.31
The reasons for not willing to continue to work in the primary health care institutions. (*n* = 98)		
The low income and fewer good policy or the policy is not been implemented	83	84.69
The limited career development	88	89.80
The poor primary medical environment	66	67.35
The imperfect general practice service mode	78	79.59
The wide range of specialties of general practice	31	31.63
The low support of primary health care institutions’ leader for GPs	32	32.65
The main work is about public health but the clinic knowledge is less used	32	32.65
The big difference with schoolmates of other specialties	15	15.31
The plan of not continuing to work in the primary health care institutions. (*n* = 98)		
Take part in the graduate candidate test	68	69.39
Find a better position	73	74.49
Practice medicine individually	21	21.43
Change profession	27	27.55

### The satisfaction of residents of GPs on standardized training

As shown in Table 3, 104 (39.54%) residents were satisfied with monthly income, and other 55 (20.91%) were unsatisfied, of whom, 30 (54.55%) thought the satisfactory monthly income was between 3500 and 4000RMB. Among all the residents, more than half (62.74%) were satisfied with teachers of standardized training, while 28 (10.64%) were unsatisfied, of whom, 25 (89.29%) were unsatisfied with teaching approach and 23 (82.14%) teaching motivation. As for six-month community training, 98 (37.26%) residents were satisfied and 76 (28.90%) were unsatisfied, mainly due to the few patients (69.74%) and the poor teaching motivation of teachers (59.21%).

**Table 3:** The satisfaction of residents of GPs on standardized training

Statements	Frequency	Percent (%)
The satisfaction of monthly income during the standardized training for residents of GPs. (*n* = 263)		
Satisfied	104	39.54
Neutral	104	39.54
Unsatisfied	55	20.91
The satisfied monthly income (RMB) of residents of GPs. (*n* = 55)		
< =3500	5	9.09
3500–4000	30	54.55
4000–4500	14	25.45
> 4500	6	1.81
The satisfaction of the teacher of standardized training for residents of GPs. (*n* = 263)		
Satisfied	165	62.74
Neutral	70	26.62
Unsatisfied	28	10.64
The unsatisfied sides for the teacher of standardized training for residents of GPs. (*n* = 28)		
Medical ethics	2	7.14
Clinical operational skill level	20	71.43
Title of teacher	18	64.29
Education of teacher	15	53.57
Teaching motivation	23	82.14
Teaching approach	25	89.29
The satisfaction of the six-month community training. (*n* = 263)		
Satisfied	98	37.26
Neutral	89	33.84
Unsatisfied	76	28.90
The unsatisfied sides for the six-month community training. (*n* = 76)		
The community training time is long	35	46.05
The few patients in community	53	69.74
The imperfect community equipments	43	56.58
The insufficient community teachers	37	48.68
The low ability of community teachers	41	53.95
The poor teaching motivation of community teachers	45	59.21

### Work situation and intention of GPs

As shown in Table 4, 36 (13.69%) had no jobs and 227 (86.31%) residents had jobs after the standardized training, including 98(43.17%) in hospitals and 129 (56.83%) in primary medical and health institutions. Around 112 (59.34%) residents received the monthly income between 3000 and 5000RMB and 95 (41.85%) residents had lower income. Only 95 (41.85%) medical institutions set up the general medical disciplines. Among those who had jobs, 9 (3.96%) residents didn’t pass the qualification of practicing medicine, 68 (29.96%) residents were registered as GPs and 150 (66.08%) were not registered for the following reasons:. “the medical institutions and sanitary bureau didn’t know the general medicine policy and didn’t agree” (53.33%), “there was no difference in the detail work contents between GPs and other doctors” (32.67%), “the diagnosis and treatment mode in the primary health care institutions was not suitable to the development of GPs” (28.00%) and “the residents didn’t want to register and liked to engage in specialized medical direction” (19.33%) Most residents (107, 71.33%) hoped that the range of practice medical license of GPs was expanded.

**Table 4:** Work situation and intention of GPs

Statements	Frequency	Percent (%)
Whether you have a job.		
Yes	227	86.31
No	36	13.69
The type of medical institutions. (*n* = 227)		
Hospital	98	43.17
Primary medical institutions	129	56.83
The hospital attributes. (*n* = 98)		
General hospital	81	82.65
Specialized hospital	8	8.16
Hospital of traditional Chinese and western medicine	9	9.18
The monthly income (RMB). (*n* = 227)		
< =3000	95	41.85
3000–5000	112	59.34
5000–7000	13	5.73
> 7000	7	3.08
The medical institutions set up general medical discipline. (*n* = 227)		
Yes	95	41.85
No	132	58.15
Whether you are registered as a GP. (*n* = 227)		
Yes	68	29.96
No	150	66.08
Without qualification of practicing medicine	9	3.96
The reasons for not been registered as GPs. (*n* = 150)		
The residents do not want to register and like to engage in specialized medical direction	29	19.33
The medical institutions and sanitary bureau do not know the general medicine policy and do not agree	80	53.33
The diagnosis and treatment mode in the primary health care institutions is not suitable to the development of GPs	42	28.00
There is no difference in the detail work contents between GPs and other doctors	49	32.67
Whether you hope to expand the range of practice medical license of GPs. (*n* = 150)		
Yes	107	71.33
No	43	28.67
Whether you are assigned department in the medical institutions. (*n* = 227)		
Yes	134	59.03
No	93	40.97
The type of assigned departments. (*n* = 134)		
GP department	19	14.18
Internal department	48	35.82
Surgery department	27	20.15
Gynecology and pediatric department	24	17.91
Other departments	16	11.94
The routine work in the GP department. (*n* = 19)		
Prevention and healthcare	5	26.32
Diagnosis and treatment of common diseases and frequently-occurring diseases and the referral of patients	13	68.42
Patients’ rehabilitation	3	15.79
Chronic disease management	8	42.11
Health management	8	42.11
Other position	7	36.84
The routine work of GPs are the same as other doctors. (*n* = 115)		
Yes	50	43.48
No	65	56.52
Whether you want to be GPs. (*n* = 115)		
Yes	62	53.91
No	53	46.09

Around 134 (59.03%) residents were assigned to the medical institution with only 19 (14.18%) in the GP department for “diagnosis and treatment of common diseases and frequently-occurring diseases and the referral of patients” (68.42%), “chronic disease management” (42.11%) and “health management” (42.11%), whereas the remaining 115 working in other departments such as internal, surgery, gynecology and pediatric departments. Of the remaining 115 residents, 50 (43.48%) did the same work as those in GP departments and 62 (53.91%) showed they wanted to be GPs.

### The job satisfaction of GPs and attitude of colleagues and local civilians to GPs

As shown in Table 5, 69 (30.40%) residents were satisfied with their job while 71 (31.28%) were unsatisfied. Only 34 (14.98%) residents were satisfied with their monthly income. Of the 102 residents unsatisfied with the income, 58 (56.86%) showed the satisfactory monthly income was between 5000 and 6500RMB. Around 94 (41.41%) indicated that their leaders put the construction of general medicine and cultivation of GPs in an important position. A low percentage (18.94%) of residents showed their colleagues knew the standardized training for residents of GPs. Only 15 (6.61%) residents thought that the local civilians knew the standardized training for residents of GPs in the primary health care institutions and only 39 (17.18%) residents thought that the local civilians recognized residents of GPs who have finished the standardized training. The main reasons for being unfamiliar with residents of GPs included that “the local civilians had no idea to the standardized training for residents of GPs” (90.38%), “the local civilians were used to visiting the familiar and experienced doctors” (78.85%), “the local civilians thought residents of GPs were too young” (69.23%) and “the local civilians didn’t recognize the medical level of the primary health care institutions” (15.38%). In order to increasing the recognition to residents of GPs who have finished the standardized training, 79.73% of residents thought that cultivation of GPs should be put in an important position and 78.85% considered that general medicine and GPs should be more publicized.

**Table 5:** The job satisfaction of GPs and attitude of colleagues and local civilians to GPs

Statements	Frequency	Percent (%)
The satisfaction of the work. (*n* = 227)		
Satisfied	69	30.40
Neutral	87	38.33
Unsatisfied	71	31.28
The satisfaction of the monthly income. (*n* = 227)		
Satisfied	34	14.98
Neutral	91	40.09
Unsatisfied	102	44.93
The satisfied monthly income (RMB). (*n* = 102)		
< =5000	15	14.71
5000–6500	58	56.86
6500–8000	23	22.55
> 8000	6	5.88
The leaders put the construction of general medicine and cultivation of GPs in an important position. (*n* = 227)		
Yes	94	41.41
No	133	58.59
The awareness of colleagues for the standardized training for residents of GPs. (*n* = 227)		
Yes	43	18.94
Neutral	90	39.65
No	94	41.41
The awareness of local civilians for the standardized training for residents of GPs in the primary health care institutions. (*n* = 227)		
Yes	15	6.61
No	212	93.39
The recognition of local civilians to residents of GPs who have finished the standardized training. (*n* = 227)		
Yes	39	17.18
Neutral	136	59.91
No	52	22.91
The reasons for being unfamiliar with residents of GPs who have finished the standardized training. (*n* = 52)		
Residents of GPs are too young	36	69.23
The local civilians are used to visiting the familiar and experienced doctors	41	78.85
The local civilians have no idea to the standardized training for residents of GPs	47	90.38
The local civilians don’t recognize the medical level of the primary health care institutions	8	15.38
Measures to increase recognition to residents of GPs who have finished the standardized training. (*n* = 227)		
The cultivation of GPs should be put in an important position	181	79.73
The health education lecture and the free diagnosis activities should be carried out	133	58.59
To increase contract services for family doctors	120	52.86
To increase publicity of general medicine and GPs	179	78.85
To increase construction of primary medical units and the quality of manager	35	15.42

## Discussion

The survey found that the overall qualified rate of standardized training for residents of GPs is high. Most residents believed that it was necessary to carry out standardized training for residents of GPs. At the same time, residents believed that their clinical ability and medical theory knowledge were improved through standardized training.

However, residents’ satisfaction with training, job and income was low. Residents of GPs were not highly motivated by an internal drive, and especially for those married, the low income created a lot of stress in their life. Among the rural order-oriented medical graduates in 2017 and 2018, half expressed their reluctance to stay at the primary health care institutions after their service was finished, more than half said that they planned to take the graduate exam or find a better job, and even someone wanted to leave the medical position. They thought that, in the primary health care institutions, the income was low, most policies were poor, some good policy was not implemented well, the career development was limited and the GP service mode was imperfect.

Among the physicians who completed the standardized training of GPs, 13.68% had no jobs. More than half of the residents worked in the primary care institutions. It also found that only 41.85% of the institutions had general medical or general outpatient clinics, and only 29.96% of the residents were registered as GPs in their institutions. There are some reasons why residents are not registered as GPs: “local institution and the health bureau did not know the specific policies of the GP”, “the current working scope of the GPs was the same as other professional residents”, and “some residents want to engage in specialist medical care”.

Only 18.94% of residents believed that their colleagues understood the standardized training for residents of GPs, and more than half of the residents believed that the institution did not pay attention to the construction of general medicine and the training of GPs. The residents’ overall satisfaction with the current work and the current job income were not high. Residents expressed that local people did not understand the standardization training for residents of GPs for they were accustomed to visiting familiar and experienced doctors and these residents were too young to be trusted for the service capabilities of primary care units.

In Gansu Province, few studies have examined the effectiveness of standardization training for residents of GPs. One study indicated that the current primary health care doctors were poor in knowledge and service capabilities, and in order to achieve the desired objective, bold reforms in the primary health care system should be carried out in the primary health care system and the welfare of primary health care doctors should be substantially improved [16]. Some studies indicated that the only ways to implement graded medical care and the key to promote primary health care were strengthening the standardized training and assessment of GPs and improving the service capacity of primary medical institutions [17–19]. Moreover, to ensure the quality of training and improve residents’ motivation and self-regulation abilities, self-directed learning (SDL) should be advocated, faculty development programs should be organized, and strong academic basis and evidence-based guidelines should be developed [20]. It is well-known that the doctors with the excellent clinical ability do not necessarily teach students well, and therefore, it is necessary to improve the teaching level of teachers, especially the training methods. One study showed it very useful to explore the applications of the instructor-based teaching method in training [21]. GPs mainly served in the primary health care institution after 3 years of training. Hence, one-sixth of the training should be implanted in the community. However, some residents may have gained experience as a junior doctor or transferable work experience beyond the medical field before. Thus, trainees differ in competencies prior to residency resulting in a different training period [20]. Literatures also suggested efforts in exploring the collaborative co-construction model of the standardized training for residents of GPs in the clinical and community bases [22]. Comparing with the primary care systems in Brazil, universal care, human rights, social justice and health equity should also be emphasized during the standardized training [23].

According to response of Ministry of Education of the People’s Republic of China to recommendation no. 5382 of the second session of the 13th National People’s Congress, general practice medical education and clinical practice was claimed for all medical undergraduates. From the perspective of international experience and the growth law of medical talents, the undergraduate education of medical colleges and universities generally sets up clinical medicine specialty, so as to lay a solid and generous medical foundation for clinical medical students. The training of GPs is mainly completed after graduation, such as standardized training of resident physicians [24]. Even in the UK with its relatively well-established model of general practice, there is still bias in medical schools towards hospital medical careers. Depending on the foreign experience, there is urgent need to develop and promote awareness and understanding of general practice for medical undergraduates, improve access to the quality of work experience in general practice for prospective medical students, and strengthen collaboration to raise the academic profile and future vision of general practice and tackle the tensions which surround general practice [25]. This will take a long time and must start as early as possible in medical training.

### Strengths and limitations

The standardized training for residents was initiated in 2014 In Gansu, China, this current study investigated the training situation, working situation and satisfaction of resident of GPs who have finished standardized training for the first time. There are some limitations in the current study that should be addressed. First, the survey was conducted soon after the first two cohorts residents of GPs graduated from standardized training, and therefore, the satisfaction was low. Presently, satisfaction should be increased over years of working in gathering the experience and improving the better treatment. The further survey will be carried out around 3 years later. In addition, in the study, there was no special survey on primary medical workers and local civilians for GPs and related policy, so the understanding of primary medical workers and local civilians for GPs and related policy was not accurate.

## Conclusions

The satisfaction for standardized training of GP residents with regard to job and income was low in Gansu. The diagnosis and treatment model in current primary care institutions is in a transitional stage in China and the standardized training for residents of GPs is increasingly improved. So the current study provides evidence and decision-making basis for cultivation of GPs. The better policies for GPs should be further formulated and executed, concerning the income of GPs, attractiveness to GPs, the publicity of standardized training, the training quality and the construction of primary medical institutions. And also the local government should make efforts to solve the employment of GPs madding them play an important roles in the primary health care.

## Data Availability

The data that support the findings of this study are not publicly available because they contain information that could compromise research participant privacy. Under certain circumstances, the data may be available from the corresponding author on reasonable request.

## Abbreviations

GPs: General Practitioners
GP: General Practice
SDL: Self-directed learning
